# Identification of a Novel Marine Fish Virus, Singapore Grouper Iridovirus-Encoded MicroRNAs Expressed in Grouper Cells by Solexa Sequencing

**DOI:** 10.1371/journal.pone.0019148

**Published:** 2011-04-29

**Authors:** Yang Yan, Huachun Cui, Songshan Jiang, Youhua Huang, Xiaohong Huang, Shina Wei, Weiyi Xu, Qiwei Qin

**Affiliations:** 1 State Key Laboratory of Biocontrol, School of Life Sciences, Sun Yat-sen University, Guangzhou, China; 2 Key Laboratory of Marine Bio-resources Sustainable Utilization, South China Sea Institute of Oceanology, Chinese Academy of Sciences, Guangzhou, China; University of Kansas Medical Center, United States of America

## Abstract

**Background:**

MicroRNAs (miRNAs) are ubiquitous non-coding RNAs that regulate gene expression at the post-transcriptional level. An increasing number of studies has revealed that viruses can also encode miRNAs, which are proposed to be involved in viral replication and persistence, cell-mediated antiviral immune response, angiogenesis, and cell cycle regulation. Singapore grouper iridovirus (SGIV) is a pathogenic iridovirus that has severely affected grouper aquaculture in China and Southeast Asia. Comprehensive knowledge about the related miRNAs during SGIV infection is helpful for understanding the infection and the pathogenic mechanisms.

**Methodology/Principal Findings:**

To determine whether SGIV encoded miRNAs during infection, a small RNA library derived from SGIV-infected grouper (GP) cells was constructed and sequenced by Illumina/Solexa deep-sequencing technology. We recovered 6,802,977 usable reads, of which 34,400 represented small RNA sequences encoded by SGIV. Sixteen novel SGIV-encoded miRNAs were identified by a computational pipeline, including a miRNA that shared a similar sequence to herpesvirus miRNA HSV2-miR-H4-5p, which suggests miRNAs are conserved in far related viruses. Generally, these 16 miRNAs are dispersed throughout the SGIV genome, whereas three are located within the ORF057L region. Some SGIV-encoded miRNAs showed marked sequence and length heterogeneity at their 3′ and/or 5′ end that could modulate their functions. Expression levels and potential biological activities of these viral miRNAs were examined by stem-loop quantitative RT-PCR and luciferase reporter assay, respectively, and 11 of these viral miRNAs were present and functional in SGIV-infected GP cells.

**Conclusions:**

Our study provided a genome-wide view of miRNA production for iridoviruses and identified 16 novel viral miRNAs. To the best of our knowledge, this is the first experimental demonstration of miRNAs encoded by aquatic animal viruses. The results provide a useful resource for further in-depth studies on SGIV infection and iridovirus pathogenesis.

## Introduction

Iridoviruses are large-genome DNA viruses infecting only invertebrates and poikilothermic vertebrates [1]. In recent years, iridoviruses have gained more attention because of the high mortality and serious systemic diseases that they cause in aquaculture. Since the first discovery of iridovirus in 1954, more than 100 iridoviruses have been isolated and classified into five genera [1], [2]. Singapore grouper iridovirus (SGIV) was first isolated from the diseased brown-spotted grouper, *Epinephelus tauvina*, and was characterized as a novel member of the genus *Ranavirus*, family *Iridoviridae* [3], [4]. SGIV has caused significant economic losses to grouper aquaculture in China and Southeast Asia. The SGIV genome sequence has been determined [5], and the whole genome transcriptional profiles of SGIV have also been mapped in virus-infected grouper spleen cells and in virus-infected grouper spleen tissues [6]. However, information about SGIV-encoded gene function and molecular mechanisms of viral pathogenesis is still limited [7]–[10]. Ranaviruses, such as frog virus 3 (FV3), the typical species of the genus *Ranavirus*, have been reported to initiate the first stage of genome replication in the nucleus and then finish self-assembly in the cytoplasm [1]. Similar to FV3, SGIV could replicate and propagate well in established fish cell lines [3]. Therefore, the features of SGIV raise the possibility to study the contribution of viral microRNAs (miRNAs) to promote SGIV pathogenesis both *in vitro* and *in vivo*. However, whether miRNAs exist in the SGIV genome remains largely unknown.

miRNAs represent a class of ∼22-nucleotide (nt) small RNA molecules that can regulate mRNA expression at the post-transcriptional level by degradation or translational repression [11]. miRNAs are encoded by a diverse range of metazoan eukaryotes and plant species, and play important roles in a wide spectrum of biological processes such as embryonic development, cell differentiation, apoptosis, and oncogenesis [12]. To date, miRNAs are also known to be produced by several viruses. Except for miRNAs in human immunodeficiency virus 1 [13], attention is increasingly being focused on miRNAs derived from DNA viruses [14]–[18]. They are mainly from the herpesvirus family, including Marek's disease virus [19], [20], herpes simplex virus (HSV)-1 and HSV-2 [21], [22], human and murine cytomegalovirus (hCMV and mCMV) [23]–[25], Kaposi's sarcoma-associated herpesvirus (KSHV) [26], [27] and Epstein-Barr virus (EBV) [28], [29], and also from simian polyomaviruses [30], as well as human adenovirus [31]. However, information about exact function of most viral miRNAs is very limited. They are characterized in facilitating virus propagation and pathogenesis by suppressing the expression of viral or cellular genes [16], [18].

To date, several studies have found that viral transcripts are regulated by viral miRNAs through perfect or imperfect matches. For example, hCMV-encoded miR-UL112-1 is capable of targeting viral immediate-early gene 1, which indicates that miR-UL112-1 plays an essential role in maintenance of the latency infection state [32]. Another instance arises from HSV-2-encoded miRNAs, which help to establish the latent viral infection state via inhibition of expression of HSV-2 ICP34.5 and ICP0 [22], [33]. In addition to targeting viral transcripts, recent studies have shown that virus-encoded miRNAs can also regulate cellular mRNA expression to evade host antiviral immune responses. The first recognized cellular mRNA targeted by EBV miR-BART5 is the mRNA of p53 upregulated modulator of apoptosis (PUMA), and inhibition of PUMA by miR-BART5 can protect EBV-infected cells from apoptosis [34]. Above all, viral miRNAs are capable of targeting viral or cellular transcripts to establish a beneficial environment for virus reproduction. Therefore, we hypothesize that blocking the viral miRNAs might enhance the host immune response to virus infection, which suggests that viral miRNAs might be applied as candidate therapeutic targets.

Recently, the second-generation sequencing platform has been developed, and the new high-throughput sequencing strategies have been used to uncover novel viral miRNAs [19], [35]–[37]. Compared with the traditional methods, the next generation sequencing strategies have been revolutionized to permit investigation of low-abundance and non-conserved novel miRNAs in a wide spectrum. In the present study, to gain further insights into the roles that miRNAs played during SGIV infection, we applied Solexa deep-sequencing technology, combined with computational techniques, to identify novel miRNAs encoded by SGIV, and the candidate viral miRNAs were further validated by stem-loop quantitative RT-PCR and luciferase reporter assay. To the best of our knowledge, the present study provides a first comprehensive genome-wide view of miRNA production for aquatic animal viruses, and these data could contribute to understanding the mechanism of iridovirus pathogenesis.

## Methods

### Cells and virus

Grouper embryonic (GP) cells [3] and fathead minnow (FHM) cells [38] were grown in Eagle's minimum essential medium that contained 10% fetal bovine serum (Invitrogen, Carlsbad, CA, USA) at 25°C. SGIV (strain A3/12/98) was originally isolated from diseased brown-spotted grouper, *E. tauvina*, and the propagation of SGIV was performed as described previously [3]. Virus was inoculated onto confluent monolayers of GP cells at an MOI of ∼0.1. When the cytopathogenic effect was sufficient, the medium that contained SGIV was harvested and centrifuged at 3000× *g* for 10 min at 4°C, and the supernatant was collected as the SGIV solution and stored at −80°C until use.

### RNA isolation and Solexa sequencing

GP cells were inoculated into three cell culture flasks (25 cm^2^) at 25°C. After allowing the cells to adhere for 18 h to 80% confluence, they were infected with SGIV at an MOI of ∼0.1. At 6 h, 24 h and 48 h post infection (p.i.), SGIV-infected cell cultures were harvested and pooled. Total RNA was extracted using TRIzol reagent (Invitrogen), according to the manufacturer's protocol, with a little modification. After addition of isopropanol, the RNA extract was incubated at −20°C for 2 h to acquire more low-molecular-weight RNAs. The isolated RNA was digested with TURBO DNase™ (Ambion, Austin, TX, USA) to discard the DNA contaminant, according to the manufacturer's protocol. The quality and integrity of the total RNA was evaluated by electrophoresis on 1.2% agarose gel and Agilent 2100 BioAnalyzer (Agilent Technologies, Santa Clara, CA, USA). The sequencing procedure was carried out as previously described [39]. Total RNA was electrophoresed on 15% polyacrylamide–8 M urea gel, and small RNAs with <30 nt were extracted from the gel and purified. Sequentially, a pair of Solexa proprietary adaptors was ligated to their 5′ and 3′ ends, followed by reverse transcription and amplification by PCR using a pair of primers complementary to the linker sequences. The generated cDNA library was utilized for sequencing analysis using the Illumina Genome Analyzer (Illumina, San Diego, CA, USA), according to the manufacturer's instructions.

### In silico analysis

Raw sequencing data were filtered by eliminating low quality reads and adaptor contaminants to generate usable reads with size ≥18 nt. The raw data and processed data were deposited into the NCBI Gene Expression Omnibus (GEO) database. A large number of small RNAs were cellular in origin, therefore, the Solexa data was aligned against zebrafish genome using SOAP v1.11 (Short Oligonucleotide Alignment Program) (http://soap.genomics.org.cn) [40], which was downloaded from the UCSC Genome Browser Database. Selecting zebrafish as a reference was attributable to the fact that zebrafish, being a model vertebrate with a sequenced genome, has a closer evolutionary relationship to grouper, compared with other model species. Sequences with perfect match were utilized for further analysis. To discard unique sequences that originated from rRNA, tRNA, small nuclear RNA (snRNA) and small nucleolar RNA (snoRNA), the retained sequences were mapped to Rfam 9.0 and NCBI Genbank database. Moreover, small RNAs derived from highly repeated elements were annotated as repeat-associated small RNAs. To identify conserved miRNAs between grouper and zebrafish, the unique small RNA sequences were analyzed by BLAST search against miRBase v12.0 (http://www.mirbase.org/). Only sequences with perfect match were considered to be conserved miRNAs.

After analysis of the cellular small RNAs, all of the clean small RNAs were aligned against the SGIV genome using SOAP v1.11, which was also downloaded from the UCSC Genome Browser Database. Only sequences that perfectly matched the SGIV genome along their entire length were considered for further analysis. The mapped sequences were blasted against known miRNAs deposited at miRBase to find conserved miRNAs. Subsequently, to understand better the secondary structures of the potential small RNAs, ∼100 nt of genomic DNA sequence flanking each side of these small RNA sequences were selected, and the ability to form characteristic hairpin structures were predicted using Mfold [41] and analyzed by MIREAP (http://sourceforge.net/projects/mireap/). Stem-loop hairpins that met the following criteria were considered typical precursors: dominant mature sequences ranging from 20 to 24 nt; secondary structures of hairpins with free-folding energy lower than −18 kcal/mol and a minimum of 14 paired, as well as not more than five asymmetry within the miR/miR* duplex. Only the sequences that fulfilled all of the criteria described above were determined as putative candidate novel viral miRNAs.

### Stem-loop quantitative RT-PCR

#### Reverse transcriptase reactions

Total RNA was extracted from SGIV-infected GP cells (6, 24 and 48 h p.i.) and corresponding uninfected cells by TRIzol reagent (Invitrogen). After that, the isolated RNA was digested with TURBO DNase™ (Ambion) to discard the DNA contaminants. The RNA concentration was quantified by Nanodrop 2000 Spectrophotometer (Thermo Scientific, Wilmington, DE, USA). Custom reverse transcriptase (RT) primers for detection of each miRNA, with a stem-loop sequence to enhance the binding specificity for mature miRNA, were designed by Applied Biosystems (Foster City, CA, USA) against the most common isoform. Each RT reaction contained 500 ng total RNA, 3 µl 5× RT primer, 0.15 µl dNTPs (100 mM), 1 µl MultiScribe™ Reverse Transcriptase (50 U/µl), 1.5 µl 10× Reverse Transcription Buffer, 0.19 µl RNase inhibitor (20 U/µl), and sterilized RNase-free water was used to adjust the total volume of the reaction mix to 15 µl. Reactions were incubated in a Bio-Rad Thermocycler (Bio-Rad, Hercules, CA, USA) at 16°C for 30 min, 42°C for 30 min, 85°C for 5 min, and then held at 4°C. The reverse-transcribed cDNA of each reaction was diluted 5-fold with sterile water and used as template in real-time quantitative PCR reactions.

#### Stem-loop quantitative PCR

To validate the expression of viral miRNAs in SGIV-infected samples, real-time quantitative PCR based on TaqMan MicroRNA assay was performed using the LightCycler® 480 Detection System (Roche, Basel, Switzerland) using TaqMan® microRNA Assays kit (Applied Biosystems). Each reaction consisted of 0.665 µl of product from the diluted RT reaction, 1× TaqMan® Universal PCR Master Mix, 0.5 µl 20× TaqMan® Small RNA Assay and sterile water. The mixture was incubated in a 384-well plate at 95°C for 10 min, followed by 40 cycles of 95°C for 15 s and 60°C for 1 min. For relative quantification of each miRNA, the typical 2^−ΔΔCt^ method was employed [42], and all experimental data were normalized to the U6 gene. All reactions including no-template and RT minus controls for each miRNA were run in triplicate. All experimental data were expressed as means ± SD from three separate biological experiments. The sensitivity of the miRNA quantification scheme was also evaluated using diluted products of miRNA amplification as the template. Following serially 10-fold dilution of the templates over five orders of magnitude, real-time quantitative PCR was performed with a separate TaqMan® Small RNA Assay kit.

### Plasmids construction

#### miRNA-expressing plasmid construction

Plasmid constructs that expressed SGIV-encoded miRNAs were generated based on the pLL3.7 modification vector, which was the same as that used by Xia et al. [43]. For each recombinant pLL3.7-miR plasmid, the inserted sequence comprised the stem-loop structure and 100–200 bp upstream and downstream flanking genome sequences of the indicated miRNA, which was expected to express and process into mature miRNAs by cellular Drosha and Dicer as naturally as possible. The approximate 500-bp fragments of each miRNA precursor were amplified from SGIV genomic DNA by the primers listed in Table 1. Agarose-gel-purified PCR fragments were digested with *Bam*HI and *Xho*I (Takara, Kyoto, Japan) and subcloned into *Bam*HI and *Xho*I sites of the pLL3.7 vector, followed by confirmation through sequencing. pLL3.7 carried a green fluorescent protein tag, therefore, the transfection efficiency was assessed by fluorescence microscopy (Leica Microsystems, Wetzlar, Germany).

**Table 1:** Sequence of PCR primers used in this study.

Primer	Sequence (5′–3′)
pLL-miR-1 F	GTCCTCGAGACGCCCCCTGTGAAATTCTCAAGA
pLL-miR-1 R	GCAGGATCCGATGCACGTGGAAGAGTACAGAATG
pLL-miR-2 F	CGACTCGAGCGCACCTGCAAACACAACGACATAC
pLL-miR-2 R	CGAGGATCCCCGGGCGATACGAGATAATTACACC
pLL-miR-5 F	GCACTCGAGGCGATTTTCCCGTTCTCTACGATGA
pLL-miR-5 R	GCAGGATCCACACAAACGTCAAATGGTTATATAC
pLL-miR-6 F	CGTCTCGAGGGTATTTTTGCTCCTGCGGGTATGG
pLL-miR-6 R	AGAGGATCCGGGGAATAACCATAACTCCAGACGC
pLL-miR-7 F	ATGCTCGAGCGGCATTGGCGAACGGAAAG
pLL-miR-7 R	GCTGGATCCGGCCCCGTGCAAAATGGAATCT
pLL-miR-9 F	CCGCTCGAGCAGAGCCTTGATAAGCGTG
pLL-miR-9 R	CGCGGATCCCAGTCAGAAAGAGATGCAAACAA
pLL-miR-10 F	TCACTCGAGATCTGACGGTGAGGTGGGAGGCG
pLL-miR-10 R	CCAGGATCCCTAAGGCTACGTACATATTTTTCCA
pLL-miR-13 F	TCACTCGAGACCCACGGTCATATACACGTAAGC
pLL-miR-13 R	TCAGGATCCGCACGCTTCTCTCACCTTCAACGAC
pLL-miR-14 F	TGGCTCGAGGGTGACGAGTTCGAATGGCCCAAAT
pLL-miR-14 R	GGAGGATCCCGATACCCAACGCTTCCAACAAACA
pLL-miR-homoHSV F	CGACTCGAGCGCGCAATCCATTTTTCCTACC
pLL-miR-homoHSV R	ATTGGATCCTCCGTTCCATCGCGTCTACCAAA
pLL-SV-S1 F	TGCCTCGAGAGTAAAACCTCTACAAATGTG
pLL-SV-S1 R	CTGGGATCCAGCCAGGAAAATGCTGATAAAAA

#### Sensor plasmid construction

To investigate whether these viral miRNAs were indeed functional, a dual-luciferase reporter vector, which had four tandem repeats of a sequence perfectly complementary to each miRNA in the 3′ untranslated region (UTR) of the *Renilla* gene, was constructed. The dual-luciferase reporter control vector, termed as modified psiCHECK-2 (psiCHECK-2M), was made based on the psiCHECK™-2 vector (Promega, Madison, WI, USA) following the method described by Voorhoeve et al. [44]. Each sensor vector was constructed by four oligonucleotides called sense1 (s1), antisense1 (as1), sense2 (s2) and antisense2 (as2) (Table S1), of which s1 and s2 both contained two copies of a sequence that was perfectly complementary to the indicated miRNA and one or two linker sequences, whereas as1 and as2 contained complementary sequences to s1 and s2, respectively. s1/as1 and s2/as2 were annealed individually after phosphorylation of s2 and as1, followed by conjunction with each other by T4 DNA ligase (Takara). The conjunct fragments were subcloned into the psiCHECK-2M in which *Bam*HI and *Xho*I sites were introduced so that the combined sequences were unidirectionally inserted downstream of the *Renilla* luciferase gene. All of the recombinant plasmids were transformed into *Escherichia coli* DH5α and subjected to nucleotide sequencing.

### Luciferase reporter assay

Transient transfection was performed using Lipofectamine 2000 reagent (Invitrogen), according to the manufacturer's protocol. FHM cells cultured in 96-well plates for 16–18 h were transiently transfected with 120 ng miRNA-expression plasmid or pLL3.7 empty vector, along with 40 ng corresponding sensor vector. Cells that were co-transfected by pLL3.7-SV40-miR-S1 and its corresponding sensor plasmid were used as positive controls; both of which sequences were chosen as reported by Sullivan [30]. In addition, stem-loop quantitative RT-PCR was also employed to determine the expression of each miRNA derived from recombinant pLL3.7-miR vectors, using separate TaqMan® microRNA Assays kit (Applied Biosystems) on the LightCycler® 480 Detection System (Roche). At 48 h post-transfection, cells were harvested and lysed. *Firefly* and *Renilla* luciferase activities were measured consecutively with the Dual-Luciferase Reporter Assay system (Promega) on PerkinElmer 2030 Multilabel Reader (PerkinElmer Life and Analytical Science, Turku, Finland), according to manufacturer's instructions. *Renilla* luciferase activities were normalized to the constitutively expressed *Firefly* luciferase activity in all cases, and expressed as the fold stimulation relative to that of pLL3.7 empty vector transfected cells.

### Statistical analysis

All statistical data were presented as mean ± SD, followed by Student's *t* test. Differences were considered statistically significant at P<0.05.

## Results

### Overview of high-throughput sequencing of SGIV-infected GP cells

**Figure 1:**
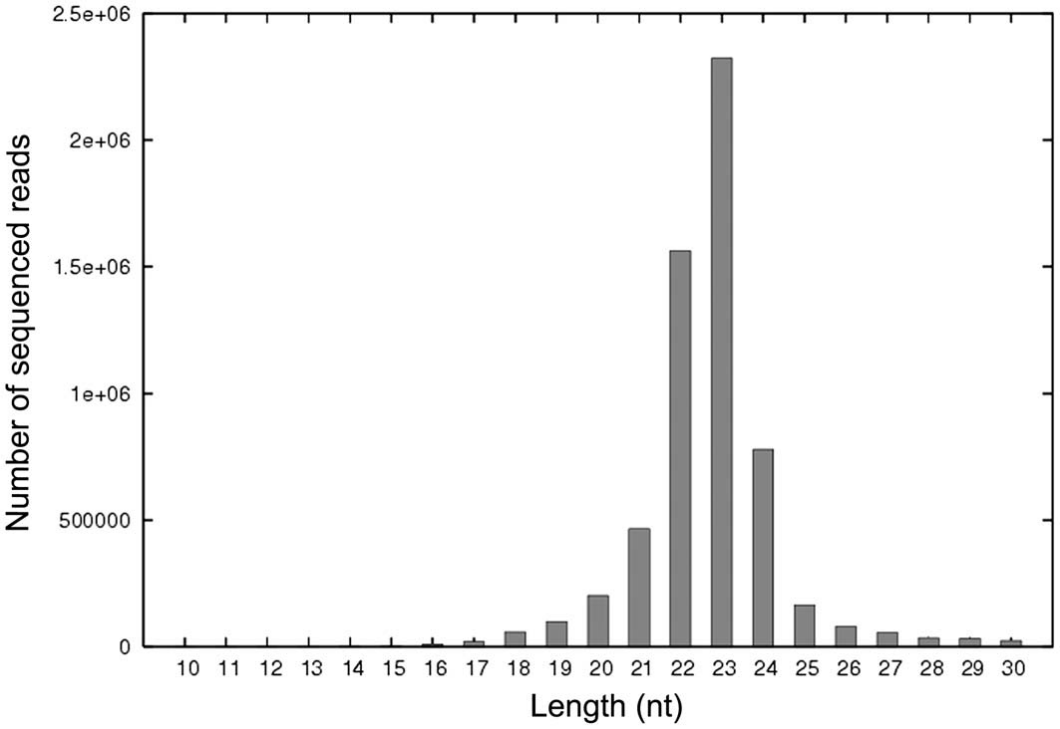
Size distribution and abundance of sequenced small RNAs from SGIV-infected GP cells. The majority of the reads were 23 nt in length.

To identify the miRNAs encoded by SGIV, a small RNA library derived from SGIV-infected GP cells was constructed and sequenced using Solexa deep-sequencing technology. A total of 7,246,099 reads were obtained, of which 6,802,977 high-quality reads were detected, which indicated 5,884,103 small RNAs. Solexa raw data and processed data are available at the NCBI GEO database under accession number GSE26909. The size distribution of sequence reads peaked at 23 nt (Fig. 1), which was consistent with the ideal size of genuine miRNAs.

Subsequently, the usable reads were aligned against the zebrafish genome with perfect match, which resulted in 3,717,379 reads matched to zebrafish genome, which indicated 18,105 unique small RNAs. These mapped small RNAs could be divided into different categories after BLAST searches against Rfam 9.0 and Genbank database, including putative fragments of rRNA, tRNA, snRNA and snoRNA (Table 2). Another fraction was derived from highly repeated elements named repeat-associated small RNAs. All of these small RNAs were abandoned, which distinguished them from the remaining RNAs for further analysis. To identify the conserved miRNAs between grouper and zebrafish, the remaining RNAs were blasted against the miRNA database, and 3,457,140 reads matched perfectly to known zebrafish miRNAs. The major fractions were attributable to the let-7 family, which consisted of let-7a, let-7b, let-7c and let-7f, among others. In consideration of the higher abundance of let-7f, we utilized it as positive control in our subsequent studies.

**Table 2:** Distribution of small RNAs from GP cells infected with SGIV.

Small RNA class	Number of reads
Total high quality reads	5,884,103
Match with zebrafish genome	3,717,379
Conserved miRNA^a^	3,457,140
rRNAetc^b^	134,305
Repeat^c^	4,585
Match with SGIV genome	34,400
Unannotation^d^	2,132,324

a Includes 138 conserved miRNAs between zebrafish and grouper fish.

b Includes rRNA, tRNA, snRNA and snoRNA.

c Represents repeat-associated small RNAs.

d Indicates sequences that do not match to SGIV or zebrafish genome.

### Bioinformatic identification of SGIV-encoded miRNAs

**Figure 2:**
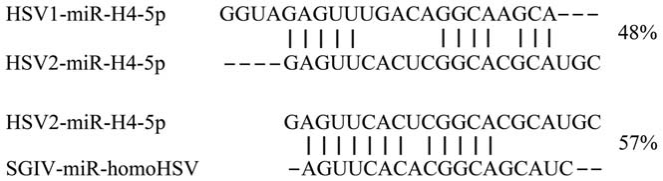
Comparison of SGIV-miR-homoHSV and conserved HSV2-H4-2-5p. Conserved nucleotides are indicated by vertical lines, and the overall percentage of sequence identities are indicated on the right.

**Figure 3:**
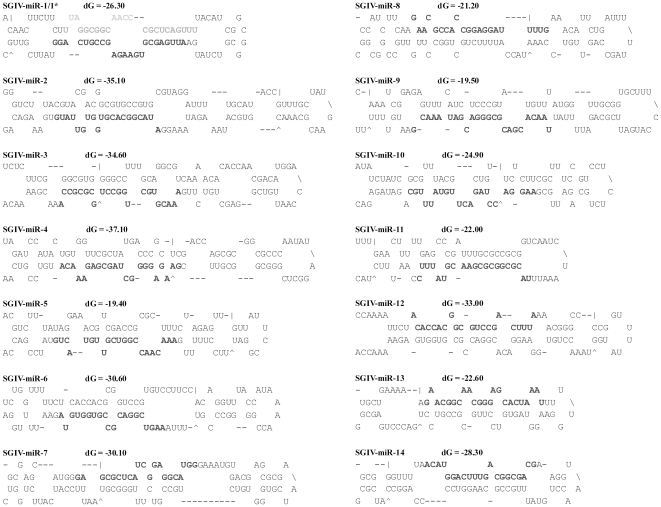
Predicted stem-loop secondary structures of SGIV-encoded candidate miRNAs. Folding and free energy calculations were determined with mfold algorithm. Dominant forms of the mature miRNAs are indicated in dark gray, and the less-abundant form of SGIV-miR-1-5p is shown in light gray.

**Figure 4:**
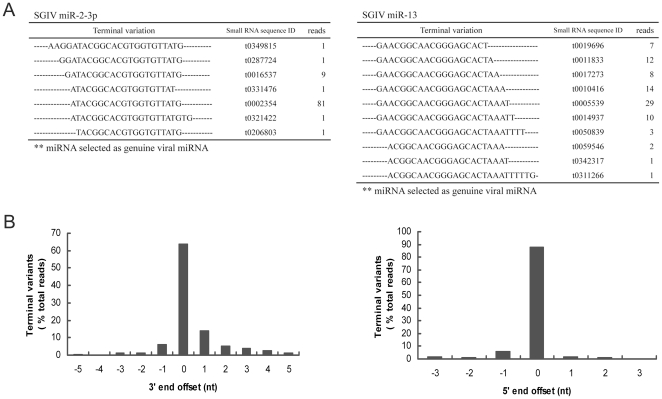
5′ and 3′ ends sequence variation of SGIV miRNAs recovered from deep sequencing. (A) Length variation of SGIV miR-2 and SGIV miR-13 are listed as examples. Distinct sequences of each miRNA, small RNA sequence ID, as well as reads number are shown. (B) Distribution of 3′ and 5′ terminal variant of SGIV miRNAs. Each unique sequence with 3′ or 5′ terminal extension corresponding to the genuine miRNA selected are appointed a negative offset number, while the unique sequence with 3′ or 5′ terminal nucleotide deletion are assigned a positive offset number. In all cases, the percentage of heterogenicity for each unique viral miRNA was obtained by dividing the read number of each variant by the total read number.

Among the 5,884,103 small RNAs obtained from deep sequencing, 34,400 reads were perfectly matched to the SGIV genome, which contained 11,144 unique small RNAs. These small RNAs were blasted against known miRNAs deposited at miRBase, and a similar sequence to HSV2-miR-H4-5p was identified (Fig. 2). We designated it SGIV-miR-homoHSV as one novel viral miRNA. After that, the SGIV genome was scanned to identify the potential hairpin region of each small RNA that was perfectly matched to the SGIV genome, using software MIREAP, and finally, a total of 15 candidate miRNAs (excepted SGIV-miR-homoHSV) derived from 14 imperfect fold-back structure precursors were identified. According to the prediction by Mfold, all the SGIV sequences flanking each candidate miRNA could be readily folded into characteristic stable hairpin structures (Fig. 3). All the above identified 15 miRNAs were designated as SGIV-miR-1 to 14, among which the miRNAs derived from the 5′ and 3′ arms of SGIV-miR-1 precursor were designated as SGIV-miR-1-5p and -3p, respectively (Table 3). Some of the SGIV miRNA sequences obtained above exhibited terminal heterogeneity at the 3′ and/or 5′ ends (Fig. 4A). Sequence terminal variation analysis revealed that the frequency of 5′ end length heterogeneity of SGIV miRNAs was approximately 12% with <3 nt deletion or extension. Conversely, the 3′ end length variation was >60% and ranged from minus 5 nt to plus 5 nt (Fig. 4B).

**Table 3:** Sequence and genomic position of SGIV miRNAs.

SGIV miRNA	Sequence (5′–3′)^a^	Length (nt)	No. of reads	Genomic position^b^
miR-1-3p	(A)ATTGAGCGTGAAGAGCCGTC(AGGTA)	20–25	24	62001–62026
miR-1-5p	(TTAGG)CGGCAACCCGCTCAGT(TTTA)	20–21	2	61959–61983
miR-2	(AAGGA)TACGGCACGTGGTGTTATG(TG)	19–24	95	109269–109294
miR-3	AAACGTGCTGGCCTGCGCGC(CA)	20–22	7	c5791–5812
miR-4	GAAGAGGGGCTAGCGAGAAACA	22	6	c21153–21174
miR-5	AAACAACCGGTCGTTGTAC(TGTA)	19–23	37	28189–28211
miR-6	(A)AGTCGGACGCCGTGGTGT(AG)	18–21	23	51458–51478
miR-7	GAGCGCTCATCGGAGGCATG(GGAA)	20–24	7	52062–52085
miR-8	(CAAG)AACGCCACCGGAGGATT(TTGAAA)	18–24	86	64167–64193
miR-9	(TTAACA)CGACGCGGGACGATA(AACGAATGT)	20–25	22	118514–118543
miR-10	(G)AAGGACCTAGACTTGTA(TTTGC)	18–23	22	133238–133260
miR-11	(TT)TACGCGGCGCGAACGTATT(TCC)	20–22	11	135009–135032
miR-12	(CT)ACACCACGGCGTCCGACTTT(A)	20–23	9	c51401–51423
miR-13	(GA)ACGGCAACGGGAGCACT(AAATTTTTG)	19–26	87	c66907–66934
miR-14	(A)CATGGACTTTGACGGCGACG	20–21	8	c97796–97816
miR-homoHSV	AGTTCACACGGCAGCATC	18	4	c119023–119040

SGIV miRNA sequences, length, number of reads and genomic positions are indicated.

a Sequence variations are indicated by variant nt in parentheses.

b Genomic positions are provided based on the published SGIV genome sequence (GenBank accession no. NC_006549).

c Complementary strand of the published SGIV genome sequence.

### Origin of SGIV-encoded miRNAs

**Figure 5:**
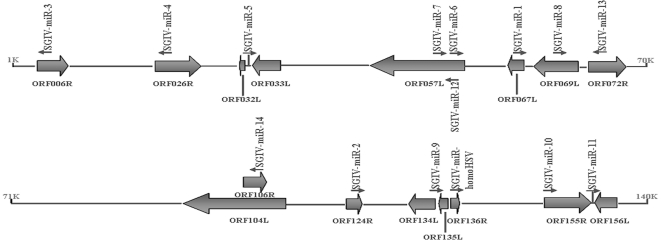
Schematic diagram of genomic location of miRNA precursors encoded by SGIV. The relative sizes, locations, and orientations of viral ORFs are shown. Small arrows indicate the transcriptional orientation and genomic position of virus-encoded miRNAs. In particular, SGIV-miR12 maps to an exonic region of ORF057L and also is antisense to SGIV-miR6. SGIV-miR5 and SGIV-miR11 locate in the intragenic region, whereas SGIV-miR9 precursor is overlapped with two ORFs, ORF134L and ORF135L.

All 16 discovered SGIV miRNAs, including the miRNA similar to HSV2-miR-H4-5p, were scattered throughout the SGIV genome (Fig. 5). In particular, three of these 16 miRNAs, including SGIV-miR-6, -7 and -12, were located within the ORF057L region of the SGIV genome. SGIV-miR-6 and -7 embedded in ORF057L in the opposite transcriptional orientation, and SGIV-miR-12 was antisense to SGIV-miR-6. Generally, localization of the remaining 13 miRNAs could be grouped into three classes that comprised lying in the same transcriptional orientation or the opposite orientation, and arising from the intragenic region. A detailed introduction is as follows: SGIV-miR-1, -3, -4, -8, -13, -14 and -homoHSV were all found to lie in the opposite orientation of the corresponding exons. Nevertheless, SGIV-miR-2 lay in the ORF124R in the same orientation, and SGIV-miR-10 in the same orientation as ORF155R. Another three miRNAs, such as SGIV-miR-5, -11 and -9, were found to arise from the intragenic region between ORF032L/ORF033L, ORF155R/ORF156L and ORF134L/ORF135L, respectively.

### Validation of SGIV miRNAs by stem-loop quantitative RT-PCR

**Figure 6:**
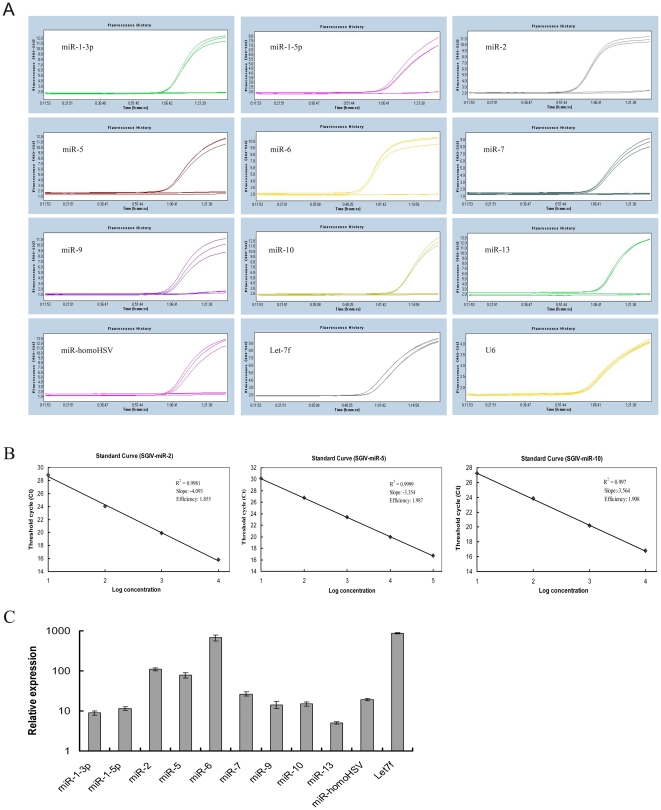
Stem-loop quantitative RT-PCR analysis of the SGIV miRNAs expression levels in SGIV-infected GP cells. (A) Amplification plot of SGIV-encoded miRNAs. (B) Standard curves of three selected SGIV miRNAs. (C) Relative expression levels of different SGIV miRNAs. All data were expressed as a ratio to SGIV-miR-13 transcripts and normalized to U6. Samples were assayed in triplicate, and mean values are displayed with SDs indicated.

To validate the authenticity of SGIV miRNAs obtained from Solexa deep sequencing, stem-loop quantitative RT-PCR was performed, using uninfected GP cells as the negative controls. Most of the candidate SGIV miRNAs could be detected in SGIV-infected cells, except SGIV-miR-3, -4, -8, -11 and -12 (Fig. 6A). However, in the negative controls, no fluorescence signal was observed. For some reason, SGIV-miR14 prevented the design of primers to detect it by stem-loop quantitative RT-PCR, as reported by Umbach et al. [45]. Compared to the expression level of let-7f that was used as the positive control, SGIV-miR-6 showed the highest expression level among all SGIV miRNAs, followed by a higher expression level of SGIV-miR-2 and SGIV-miR-5. Meanwhile, stem-loop quantitative RT-PCR results revealed that other viral miRNAs were present at different but easily detectable levels (Fig. 6C). The sensitivity of the miRNA quantification scheme was also evaluated. The data illustrated good linearity between the log of template concentration at different dilutions versus the corresponding cycle threshold, and some of the amplification standard curves were selectively shown in Fig. 6B.

### The determination of viral miRNAs potential function

**Figure 7:**
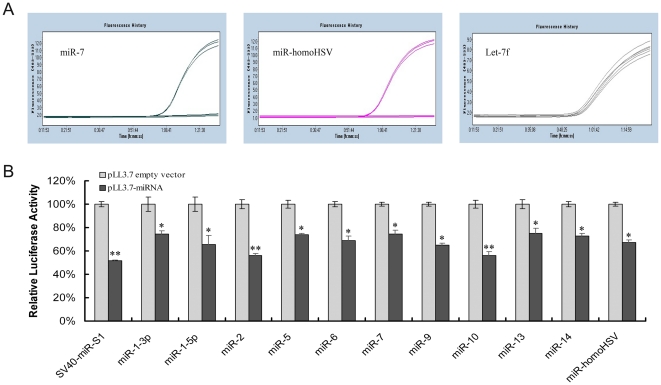
Biological activity of SGIV miRNAs. (A) Amplification curves derived from three indicated recombinant PLL3.7-miR vectors. (B) Biological activity of viral miRNAs examined by dual-luciferase reporter assay. The sensor plasmid bearing four repeats complementary to each miRNA in its 3′ UTR of *Renilla* reporter gene was co-transfected into FHM cells, along with the indicated miRNA-expressing vector. The cells co-transfected by pLL3.7-SV40-miR-S1 and the corresponding sensor plasmid were used as positive controls. After transfection for 48 h, samples were harvested and dual luciferase activities were validated. Data are expressed as a ratio to negative controls with SDs, and the significant differences are indicated with * at p<0.05 or ** at p<0.01.

To determine whether the identified viral miRNAs were indeed functional, 11 miRNA-expressing plasmids and 12 dual-luciferase reporter vectors were selectively constructed based on stem-loop quantitative RT-PCR results, and used for luciferase reporter assay. Broad expression of mature miRNAs that originated from the indicated recombinant pLL3.7-miR vectors was observed, and some amplification curves are selectively listed in Fig. 7A. Coincident with the downregulated luciferase activity of the positive control miRNA, SV40-miR-s1, all of the SGIV miRNAs that passed the validation of stem-loop quantitative RT-PCR were able to suppress the luciferase activity (>25–50%) (Fig. 7B). SGIV-miR-2 and SGIV-miR-10 significantly repressed *Renilla* luciferase activity and exerted an approximately 2-fold inhibitory effect compared to separate negative controls. In addition, SGIV-miR14 was also able to inhibit its corresponding reporter activity by 27%. Combined with the feature that SGIV-miR14 precursor can be folded into a characteristic hairpin secondary structure, we considered it as an authentic miRNA, although we could not detect it by stem-loop quantitative RT-PCR. As noted above, the results of luciferase reporter assay were consistent with those from stem-loop quantitative RT-PCR; both of which contributed to assessment of the credibility of novel SGIV miRNAs.

## Discussion

*Iridoviridae*, like *herpesviridae*, belongs to the large double-stranded DNA virus family [46], [47]. SGIV is a novel member of the genus *Ranavirus*, family *Iridoviridae*, and is a large dsDNA virus. The entire SGIV genome consists of 140,131 bp with 162 predicted open reading frames [5]. Similar to FV3, SGIV is proposed to initiate the first stage of genome replication in the nucleus and finish its self-assembly in the cytoplasm. Previous studies have shown that viral miRNA biogenesis, like cellular miRNAs, also initiates transcription of primary miRNAs in the nucleus, followed by sequential processing under two RNase IIIs Drosha and Dicer to produce an ∼22-nt RNA duplex. Finally, only one strand of the viral original duplex is incorporated into RNA-induced silencing complexes to direct the cleavage or translational inhibition of target mRNA [18], [48]. To the best of our knowledge, miRNAs encoded by aquatic animal viruses have not been experimentally identified yet.

Several methods have been carried out to determine the expression of miRNAs in previous studies, including northern blotting, cloning, and microarray assay. Northern blotting is capable of detecting pre- and mature miRNAs simultaneously, but it is not sensitive enough to validate the low-abundance miRNAs, as well as its poor throughput. The cloning approach has two major disadvantages: one is the poor throughput, and the other is that it is time consuming [49]. Microarray assay can improve sampling depth and sensitivity, however, it is restricted to the detection and profiling of known miRNA sequences previously identified by experiment or homology searches, and even yields a high frequency of non-specific signals [50]. Compared with the traditional methods, high-throughput sequencing can yield a large number of small RNAs, which raises the possibility of recovering novel miRNAs in a given sample. Thus, in this study, we applied Solexa deep-sequencing to explore the expressed miRNAs in SGIV-infected GP cells. A total of 5,884,103 effective reads were obtained, of which, 34,400 were perfectly matched to the SGIV genome. After BLAST search against the miRBase and predicting the secondary structure, 16 novel viral miRNAs were explored. However, none was detected by northern blotting (data not shown). This might have been due to the low level of spontaneous expression of these virus-encoded miRNAs, which was consistent with the low percentage of sequenced viral small RNAs divided by total small RNA reads. In addition, >100 conserved miRNAs of cellular origin in grouper and zebrafish were detected after BLAST search in the miRBase, which contributed to exploring how cellular miRNAs influence viral resistance and pathogenesis through regulation of viral or cellular gene expression.

In the present study, the identified SGIV miRNAs exhibit sequence variations frequently, and two classes of variants that comprised of 5′ and 3′ terminal variability were found, as with iso-miR [27], [50]. It is well known that the target mRNA is recognized by miRNA via its 5′ seed sequence, and the 5′ end length heterogeneity can greatly affect the ability of an miRNA to target corresponding mRNAs. In contrast, the role of the 3′ end in target discrimination is less important. Our studies revealed that SGIV miRNA sequences showed high homogenicity in their 5′ ends but common heterogeneity at their 3′ ends, which is consistent with those found in KSHV and EBV miRNAs [27], [35]. While for SGIV-miR-1-3p, -6, and -13, the 5′ end variation might be able to affect the selection of the mRNAs targeted by these viral miRNAs. Generally, the length variation for all SGIV miRNAs revealed that the frequency of 5′ end length heterogeneity was ∼12% with <3 nt deletion or extension. Conversely, the 3′ end length variation was >60% and ranged from −5 to +5 nt. It has been proposed that this phenomenon arises from the distinct cleavage by Drosha and/or Dicer, and is not due to artifacts created from sequencing [50].

In contrast to cellular miRNAs, viral miRNAs do not share a high level of homology, which could be due to the higher mutation rate and faster evolution compared with their vertebrate counterparts [51]. However, between closely related EBV and Rhesus lymphocryptovirus (rLCV), eight miRNAs are conserved at the level of mature miRNA sequences [52]. In addition, some hCMV pre- and mature miRNAs sequences, identified by bioinformatic prediction without experimental validation, also share homology with miRNAs of chimpanzee CMV [18]. In our study, a novel SGIV-encoded miRNA (SGIV-miR-homoHSV) was found to be similar to HSV2-miR-H4-5p, with mature sequences with 12 out of 18 nucleotide identity. Compared to the 48% sequence identity of miR-H4-5p between closely related HSV-1 and HSV-2, SGIV-miR-homoHSV shared 57% overall sequence identity with HSV2-miR-H4-5p. Of note, this miRNA was validated experimentally by stem-loop quantitative RT-PCR and confirmed by luciferase reporter assay, which suggests that viral miRNA conservation might exist in evolutionarily distant viral species.

To date, it is much easier to identify the viral gene target of a viral miRNA, partly because viral miRNA transcribed from the opposite strand of a viral coding gene makes an obvious target. Most reported viral miRNAs are located antisense to the protein coding genes, which results in a perfect match between miRNA and mRNA, which has implications for regulation of viral mRNA expression [17], [18]. For instance, SV40 miR-s1 targets viral early transcript T antigen with perfect matches, which results in decreased susceptibility of SV40-infected cells to cytotoxic T lymphocyte killing [30]. Barth et al. have reported EBV miR-BART2, exhibiting antisense to EBV DNA polymerase BALF5, degraded BALF5 production and might be capable of maintaining EBV latent infection [53]. In our study, nine out of 16 SGIV-encoded miRNAs were located in opposite strands to the coding genes. For instance, SGIV-miR-13 was found to lie in the opposite transcriptional orientation of ORF072R, which encodes the major capsid protein, and SGIV-miR-homoHSV was embedded in ORF136R in the opposite transcriptional orientation, which has been recently designated as the viral lipopolysaccharide-induced TNF-α factor (LITAF), and reported to contribute to virus replication by exploitation of cell apoptosis and regulation of host immune responses [7]. Previous studies have shown that three EBV-encoded miRNAs target EBV latent membrane protein 1 (LMP1), which inhibits cell growth by increasing cellular sensitivity to apoptotic stimuli. This results in attenuation of the pro-apoptotic effect of LMP1, which ensures cell survival [28]. Whether the SGIV-miR-homoHSV plays a similar anti-apoptotic role to that proposed for EBV miRNAs needs further investigation. Furthermore, until now, several anti-microRNA oligonucleotides with some modifications, such as 2′-O-methyl-substituted RNA oligonucleotides and mixed locked nucleic acid-DNA, have been presumed to be novel diagnostic and therapeutic agents [51]. Therapeutics based on SGIV-encoded miRNAs could help to deal with SGIV-caused fish viral disease.

Taken together, 34,400 SGIV-encoded candidate small RNAs were obtained by Solexa deep sequencing. Finally, a total of 16 SGIV-encoded miRNAs were identified through *in silico* analysis, of which, 11 viral miRNAs passed validation with stem-loop quantitative RT-PCR and luciferase reporter assay. Our present results provide the foundations for further studies on SGIV infection and pathogenesis mechanisms, and will also empower researchers to design novel diagnostic and therapeutic strategies to combat this aquatic animal virus.

## Supporting Information

Table S1 Oligonucleotides used in sensor plasmids construction.
(DOC)
