# Supplementary material for: Identification of a Novel Marine Fish Virus, Singapore Grouper Iridovirus-Encoded MicroRNAs Expressed in Grouper Cells by Solexa Sequencing
Source: PLoS One. 2011 Apr 29;6(4):e19148. doi: 10.1371/journal.pone.0019148 (PMC3084752; doi:10.1371/journal.pone.0019148)
Supplement: Table S1 — Oligonucleotides used in sensor plasmids construction. (DOC) [file pone.0019148.s001.doc]

**Table S1.** Oligonucleotides used in sensor plasmids construction.

| Oligonucleotides | Sequence (5'–3') |
| --- | --- |
| pSi-miR-1-3p-S1 | TCGAGTAACCTGACGGCTCTTCACGCTCAATCGATCCTGACGGCTCTTCACGCTCAAT |
| pSi-miR-1-3p-S2 | CACGACCTGACGGCTCTTCACGCTCAATTCACCCTGACGGCTCTTCACGCTCAATG |
| pSi-miR-1-3p-AS1 | TCGTGATTGAGCGTGAAGAGCCGTCAGGATCGATTGAGCGTGAAGAGCCGTCAGGTTAC |
| pSi-miR-1-3p-AS2 | GATCCATTGAGCGTGAAGAGCCGTCAGGGTGAATTGAGCGTGAAGAGCCGTCAGG |
| pSi-miR-1-5p-S1 | TCGAGTAAAAACTGAGCGGGTTGCCGCCTAACGATAAACTGAGCGGGTTGCCGCCTAA |
| pSi-miR-1-5p-S2 | CACGAAAACTGAGCGGGTTGCCGCCTAATCACAAACTGAGCGGGTTGCCGCCTAAG |
| pSi-miR-1-5p-AS1 | TCGTGTTAGGCGGCAACCCGCTCAGTTTATCGTTAGGCGGCAACCCGCTCAGTTTTTAC |
| pSi-miR-1-5p-AS2 | GATCCTTAGGCGGCAACCCGCTCAGTTTGTGATTAGGCGGCAACCCGCTCAGTTT |
| pSi-miR-2-S1 | TCGAGTAACATAACACCACGTGCCGTATCGATCATAACACCACGTGCCGTAT |
| pSi-miR-2-S2 | ACACGACATAACACCACGTGCCGTATTCACCATAACACCACGTGCCGTATG |
| pSi-miR-2-AS1 | TCGTGTATACGGCACGTGGTGTTATGATCGATACGGCACGTGGTGTTATGTTAC |
| pSi-miR-2-AS2 | GATCCATACGGCACGTGGTGTTATGGTGAATACGGCACGTGGTGTTATG |
| pSi-miR-5-S1 | TCGAGTAACAGTACAACGACCGGTTGTTTCGATCAGTACAACGACCGGTTGTTT |
| pSi-miR-5-S2 | CACGACAGTACAACGACCGGTTGTTTTCACCAGTACAACGACCGGTTGTTTG |
| pSi-miR-5-AS1 | TCGTGAAACAACCGGTCGTTGTACTGATCGAAACAACCGGTCGTTGTACTGTTAC |
| pSi-miR-5-AS2 | GATCCAAACAACCGGTCGTTGTACTGGTGAAAACAACCGGTCGTTGTACTG |
| pSi-miR-6-S1 | TCGAGTAATACACCACGGCGTCCGACTTCGATTACACCACGGCGTCCGACTT |
| pSi-miR-6-S2 | CACGATACACCACGGCGTCCGACTTTCACTACACCACGGCGTCCGACTTG |
| pSi-miR-6-AS1 | TCGTGAAGTCGGACGCCGTGGTGTAATCGAAGTCGGACGCCGTGGTGTATTAC |
| pSi-miR-6-AS2 | GATCCAAGTCGGACGCCGTGGTGTAGTGAAAGTCGGACGCCGTGGTGTA |
| pSi-miR-7-S1 | TCGAGTAACCATGCCTCCGATGAGCGCTCCGATCCATGCCTCCGATGAGCGCTC |
| pSi-miR-7-S2 | CACGACCATGCCTCCGATGAGCGCTCTCACCCATGCCTCCGATGAGCGCTCG |
| pSi-miR-7-AS1 | TCGTGGAGCGCTCATCGGAGGCATGGATCGGAGCGCTCATCGGAGGCATGGTTAC |
| pSi-miR-7-AS2 | GATCCGAGCGCTCATCGGAGGCATGGGTGAGAGCGCTCATCGGAGGCATGG |
| pSi-miR-9-S1 | TCGAGTAACGTTTATCGTCCCGCGTCGTGTTACGATCGTTTATCGTCCCGCGTCGTGTTA |
| pSi-miR-9-S2 | CACGACGTTTATCGTCCCGCGTCGTGTTATCACCGTTTATCGTCCCGCGTCGTGTTAG |
| pSi-miR-9-AS1 | TCGTGTAACACGACGCGGGACGATAAACGATCGTAACACGACGCGGGACGATAAACGTTAC |
| pSi-miR-9-AS2 | GATCCTAACACGACGCGGGACGATAAACGGTGATAACACGACGCGGGACGATAAACG |
| pSi-miR-10-S1 | TCGAGTAAGCAAATACAAGTCTAGGTCCTTCCGATGCAAATACAAGTCTAGGTCCTTC |
| pSi-miR-10-S2 | CACGAGCAAATACAAGTCTAGGTCCTTCTCACGCAAATACAAGTCTAGGTCCTTCG |
| pSi-miR-10-AS1 | TCGTGGAAGGACCTAGACTTGTATTTGCATCGGAAGGACCTAGACTTGTATTTGCTTAC |
| pSi-miR-10-AS2 | GATCCGAAGGACCTAGACTTGTATTTGCGTGAGAAGGACCTAGACTTGTATTTGC |
| pSi-miR-13-S1 | TCGAGTAAATTTAGTGCTCCCGTTGCCGTTCCGATATTTAGTGCTCCCGTTGCCGTTC |
| pSi-miR-13-S2 | CACGAATTTAGTGCTCCCGTTGCCGTTCTCACATTTAGTGCTCCCGTTGCCGTTCG |
| pSi-miR-13-AS1 | TCGTGGAACGGCAACGGGAGCACTAAATATCGGAACGGCAACGGGAGCACTAAATTTAC |
| pSi-miR-13-AS2 | GATCCGAACGGCAACGGGAGCACTAAATGTGAGAACGGCAACGGGAGCACTAAAT |
| pSi-miR-14-S1 | TCGAGTAACGTCGCCGTCAAAGTCCATGTCGATCGTCGCCGTCAAAGTCCATGT |
| pSi-miR-14-S2 | CACGACGTCGCCGTCAAAGTCCATGTTCACCGTCGCCGTCAAAGTCCATGTG |
| pSi-miR-14-AS1 | TCGTGACATGGACTTTGACGGCGACGATCGACATGGACTTTGACGGCGACGTTAC |
| pSi-miR-14-AS2 | GATCCACATGGACTTTGACGGCGACGGTGAACATGGACTTTGACGGCGACG |
| pSi-miR-homoHSV-S1 | TCGAGTAAGATGCTGCCGTGTGAACTCGATGATGCTGCCGTGTGAACT |
| pSi-miR-homoHSV-S2 | CACGAGATGCTGCCGTGTGAACTTCACGATGCTGCCGTGTGAACTG |
| pSi-miR-homoHSV-AS1 | TCGTGAGTTCACACGGCAGCATCATCGAGTTCACACGGCAGCATCTTAC |
| pSi-miR-homoHSV-AS2 | GATCCAGTTCACACGGCAGCATCGTGAAGTTCACACGGCAGCATC |
| pSi-SV40-S1-S1 | TCGAGAGTAAAACCTCTACAAATGTGGTATGGCTGATTATGATCATGAACAGACTGT |
| pSi-SV40-S1-S2 | GAGGACTGAGGGGCCTGAAATGAGCCTTGGGACTGTGAATCAATGCCTGTTTCATGCCC |
| pSi-SV40-S1-AS1 | GTCCTCACAGTCTGTTCATGATCATAATCAGCCATACCACATTTGTAGAGGTTTTACTC |
| pSi-SV40-S1-AS2 | GACTCAGGGCATGAAACAGGCATTGATTCACAGTCCCAAGGCTCATTTCAGGCCCCTCA |

.
